# (Biased) Grading of Students’ Performance: Students’ Names, Performance Level, and Implicit Attitudes

**DOI:** 10.3389/fpsyg.2018.00481

**Published:** 2018-05-09

**Authors:** Meike Bonefeld, Oliver Dickhäuser

**Affiliations:** Department of Psychology, University of Mannheim, Mannheim, Germany

**Keywords:** evaluation, teacher expectation, confirmation bias, performance, grades

## Abstract

Biases in pre-service teachers’ evaluations of students’ performance may arise due to stereotypes (e.g., the assumption that students with a migrant background have lower potential). This study examines the effects of a migrant background, performance level, and implicit attitudes toward individuals with a migrant background on performance assessment (assigned grades and number of errors counted in a dictation). Pre-service teachers (*N* = 203) graded the performance of a student who appeared to have a migrant background statistically significantly worse than that of a student without a migrant background. The differences were more pronounced when the performance level was low and when the pre-service teachers held relatively positive implicit attitudes toward individuals with a migrant background. Interestingly, only performance level had an effect on the number of counted errors. Our results support the assumption that pre-service teachers exhibit bias when grading students with a migrant background in a third-grade level dictation assignment.

## Introduction

Students with a migrant background often perform worse and achieve poorer educational results than students without a migrant background (Haycock, 2001; Lee, 2002; Dee, 2005). We know from previous large-scale studies, such as Program for International Student Assesment (PISA), that students with a migrant background more often attend lower school tracks than students without a migrant background, whereas the latter group is overrepresented in higher school tracks (Macrae et al., 1994; Lucas, 2001; Ansalone, 2003; Caro et al., 2009; Glock and Krolak-Schwerdt, 2013). Additionally, students with a migrant background drop out of school earlier than their peers without a migrant background (Rumberger, 1995). In Germany, individuals with a Turkish migrant background form the largest minority group (Statistisches Bundesamt, 2016). If we look specifically at students with a Turkish background, past research has shown that this group is overrepresented in the lower school tracks and underrepresented in higher school tracks (Baumert and Schümer, 2002; Kristen and Granato, 2007). Importantly, these differences between students with a migrant and non-migrant background remain statistically significant even after taking into account differences in scholastic performance or performance-related factors. For instance, longitudinal data collected by Dauber et al. (1996) showed that test scores strongly explained middle school placements in the United States, but differences depending on migrant background remained. Additionally, a study using data from the German micro-census showed that disparities between students of different ethnic backgrounds were explained to a great extent by prior educational attainment and social factors, but still remained statistically and practically significant after taking these variables into account (Kristen and Granato, 2007). A similar pattern was found with regard to the grading of students with and without a migrant background in mathematics. The differences were smaller but remained statistically significant after controlling for actual performance, language, and social background (Bonefeld et al., 2017). Given that the disadvantages are not only founded in a lower scholastic performance – as suggested by former research – we have to ask ourselves where the remaining differences, which depend on migrant background, come from. The differentiation between primary disparities (as a result of differences in scholastic performance or verbal skills) and secondary disparities (not related to performance) is important because the differences that remain after taking performance and related factors into account may be an indication of secondary disparities in performance assessment.

Besides being the largest migrant group in Germany, Turkish migrants are possibly one of the most important groups in terms of being disadvantaged in the school context, because past research has shown that specific stereotypes are connected with them, especially regarding their performance (Kahraman and Knoblich, 2000; Froehlich et al., 2016). These stereotypes might come into play if students’ performance is graded by a teacher.

### Role of the Teacher

Teachers interact with students, decide whether a student needs more or less support, decide on grades, and give suggestions on or even determine the type of secondary school a student attends – which is especially important in Germany and other countries with multi-track educational systems.

On a more subtle level, teachers can even change the way students perform because of the wide-known phenomenon of the *self-fulfilling prophecy* (Rosenthal and Jacobson, 1968; Jussim and Harber, 2005). Research on the self-fulfilling prophecy has demonstrated that teacher expectations (which may be based on students’ migrant background) can influence students’ behavior and their achievement. Besides these effects on students’ achievement, which may result from differences in the teacher–student interaction, information about a student’s background may also influence teachers’ perception of the student’s achievement, even though objectively there might be no differences between students. The present study is concerned with this latter effect and tests potential differences in assigned grades which depend on students’ migrant background.

Because of the many possibilities of directly and indirectly influencing students, it is obvious that teachers can contribute to the differences between students. Past research has demonstrated that teachers do not necessarily use all the information they have about a student for their judgment. It has been shown that the same performance is evaluated differently depending on various other characteristics of a student (such as social origin; Mare, 1980; Ramist et al., 1994; Walton and Spencer, 2009). Individuals who believe that a child has a higher/lower socioeconomic status rate them significantly better/worse compared to those who do not have any information about the child’s socioeconomic background (Darley and Gross, 1983). Previous research has revealed such differences for students with a migrant background and students from minorities within the United States (McCombs and Gay, 1988; Chang and Sue, 2003; Parks and Kennedy, 2007) and has demonstrated this for both service and pre-service teachers (Glock et al., 2015).

These differences in teachers’ assessment of students according to group characteristics (such as migrant background) that are not necessarily related to performance may partly be rooted in different stereotypes regarding these characteristics.

A stereotype about a group a student belongs to could provide teachers with additional information (Jussim et al., 1996). Previous research has shown that teachers in part have stereotypical attitudes, which may result in biased judgments (Dee, 2005; Parks and Kennedy, 2007; Wiggan, 2007). Even though stereotypes are widely shared, individuals may, to some degree, differ in their attitudes concerning members of different groups.

Judgmental errors could arise as a result of specific teacher attitudes toward the future performance level of students from a specific group. Analyses of cultural stereotypes in Germany have shown that Germans with a migrant background are associated less strongly with performance and success-related attributes by other Germans compared to Germans without a migrant background (Kahraman and Knoblich, 2000; Froehlich et al., 2016). Furthermore, teachers tend to judge students with a migrant background as less academically able than students without a migrant background (Glock and Krolak-Schwerdt, 2013). This could be particularly important for the assessment of students with a migrant background because negative stereotypes about their performance could lead to negative attitudes about their performance and may influence the grading process. Due to negative stereotypes regarding the lower performance potential of students with a migrant background, teachers could have additional information about the student concerning characteristics that do not exist or, at least, that cannot be observed (Taylor, 1981; Walton and Spencer, 2009; van Ewijk, 2011).

#### Stereotype-Confirming Situations as Information

One additional factor that influences biased teacher judgments could be the performance level of the students the teacher has to assess. This is because stereotype-confirming situations are more likely to activate stereotypical beliefs and, therefore, more likely to result in stereotype-based judgments and, in turn, biased judgments (Brewer et al., 1981; Bodenhausen et al., 1995; Glock and Krolak-Schwerdt, 2013).

Generally speaking, this means stereotypes should more strongly affect judgments when information is given that fits the stereotype (e.g., a poor performance level). Research has confirmed this assumption: Teachers’ judgments about a student were biased against nationality when they received stereotype-confirming information about that student (Glock and Krolak-Schwerdt, 2013; Glock et al., 2015).

However, we also assume that this proven effect depends on the type of attitude a teacher holds. Previous research did not take attitudes into account in this context. We assume that a stereotype-confirming situation is more likely to activate a stereotype if this stereotype is shared by the person. For example, a poor performance level in a dictation by a student with a migrant background is more likely to activate the stereotype that individuals with migrant backgrounds perform worse if this stereotype is shared by the teacher.

#### Role of Attitudes and Implicit Associations

We previously mentioned the role of stereotypes in the process of performance assessment. Stereotypes are broadly shared assumptions in society about certain characteristics of members of certain groups. However, even though these assumptions are broadly shared, not all individuals share all stereotypes, and persons may differ with regard to how strongly they hold these stereotypes. More specifically, the association of the members of certain groups with certain characteristics may vary according to the individual. Some individuals might have more pronounced stereotypes than others. Thus, persons such as teachers can have different associations concerning members of different groups. This variation in strength and nature of association should lead to varying stereotype-related attitudes and influences on judgments, depending on the strength of the association.

Associations are related to attitudes, which are defined as associations between objects (e.g., members of a group) and corresponding evaluations. Such associations or evaluations can be positive or negative (Fazio, 2007). Depending on the intensity of associations, attitudes are stable and important in terms of guiding behavior (Krosnick and Petty, 1995). Additionally, they can influence information processing and guide attention (Roskos-Ewoldsen and Fazio, 1992).

In this case, implicit attitudes are possibly of great importance because they are automatic evaluations (Gawronski and Bodenhausen, 2006). They can be automatically activated and are spontaneous evaluations (van den Bergh et al., 2010). The automatic activation of implicit attitudes explains why they are often beyond our control and why persons are often not even consciously aware of them (Gawronski and Bodenhausen, 2006).

In this paper, we look specifically at the role of implicit attitudes, which are especially important in terms of guiding automatic behavior and play a role when individuals have fewer cognitive resources, which might be the case for teachers who have to deal with different tasks simultaneously. Additionally because implicit attitudes are less controlled, implicit associations are considered to be less susceptible to social desirability (Steffens, 2004). This is particularly important with regard to attitudes toward individuals with a migrant background.

As already mentioned, individuals are often unaware of their implicit attitudes, which can unconsciously impact teachers’ perceptions of their students (Olson and Fazio, 2009). Previous research has shown that implicit attitudes toward students from minorities predict teachers’ non-verbal behavior and the general impression they form (Nosek et al., 2002; van den Bergh et al., 2010). All in all, this suggests that implicit attitudes can lead to biased performance assessments in the school environment. Therefore, implicit attitudes toward students with a migrant background might play a moderating role between migrant background and performance assessment (Glock and Kovacs, 2013).

#### Rule-Based Processing

Dual-process models help us to understand how other individuals might form impressions. Sloman’s (1996) dual-process model dealing with reason and problem-solving describes rule-based processing. This type of processing is said to be consciously controlled and effortful. It involves information search, retrieval, and the use of task-relevant information (Petty et al., 1981; Fiske and Neuberg, 1990; Sloman, 1996). Such judgments therefore mostly follow formal rules and focus on the given task (Smith and DeCoster, 1999).

To transfer this to our study, it is important to mention why and when we use stereotypes for making judgments. Information that complies with a stereotype can be used to close knowledge gaps and help to form a judgment. However, closing knowledge gaps is not always necessary when making judgments. Such gaps can, for example, arise in terms of judgments, when teachers have to give a comprehensive assessment of a student. These judgments can be, in essence, rule-based because they rely on rule-based judgments (such as counting errors) but the rule-based judgments have to be transferred into comprehensive statements. Such a judgment, which is less rule based, consists of an overall judgment (for example, a grade) that takes different (possibly rule-based) information into account (e.g., number of errors, type of errors, grading standards). This transformation might follow fewer, less clear rules (e.g., in terms of weighting several factors) than a judgment about only one factor. Stereotypes are less likely to influence more rule-based ratings, where only a small number of conclusions need to be drawn (Biernat and Manis, 1994). Such judgments (as indicated by error numbers) can be narrowed down by clear rules, without having to integrate or evaluate different information (Smith and DeCoster, 1999). Therefore, we can assume that additional (stereotype-based) information has less influence in this latter case.

Grades represent an important factor for school success, but as past research has shown, there are no error-free assessment tools (Geiser and Santelices, 2007). They are an important example of a less rule-based judgment or a judgment in which precise formal rules do not exist. In contrast, clear rule-based judgments, such as counting errors or assessing an answer as *right* or *wrong*, should be less biased. Therefore, it is interesting to compare these two types of judgments in terms of the occurrence and the size of judgmental errors.

### Overview of the Study

In the present study, we tested whether teachers’ assessment of students’ performance (e.g., the grade given for a dictation) depends on students’ migrant background, the actual performance level of the student, and on the teachers’ implicit attitude.

We experimentally investigated whether the same performance in a dictation of a student with a (supposedly) Turkish migrant background was assessed worse than a student who appeared to be of German origin. Furthermore, we varied the quality of the performance. Half of the test persons assessed an average performance by a student who appeared to have either a migrant or non-migrant background, and the other half graded a poor performance by a student who appeared to have either a migrant or non-migrant background. The idea behind this was to also test for the main effects of performance level. By additionally assessing teachers’ implicit attitude, we aimed to test whether biased grading of students with a migrant background was more pronounced when the students performed poorly and when teachers held rather negative attitudes.

Teachers assigned a grade for the dictation (a less rule-based judgment) and also counted the number of errors (a rule-based judgment). This allowed us to test whether the biased performance assessment was more pronounced for less rule-based judgments than for rule-based judgments.

In summary, this study examined whether there were differences in grades and errors between students with or without a migrant background, and whether these differences depended on the performance level and the implicit attitude of the person assessing the performance.

## Hypotheses

In this work, an experimental design is used to investigate the mechanisms by which the disparities students with migrant background have to face arise as a result of differences in the grading processes. For this, we looked at students’ dictation performance in terms of the assigned grade and at the number of errors in the dictation. More specifically, we tested whether pre-service teachers’ assessment of students’ performance (e.g., the grade assigned for a dictation) depended on students’ actual performance level, their migrant background, and on the pre-service teachers’ implicit attitude toward individuals with a migrant background.

For the dependent variable *grade*, we expected a statistically significant main effect for performance level. Poor performance in the dictation should be rated worse than average performance. Furthermore, a statistically significant main effect of a migrant background status was assumed. We assumed that the dictation by a student believed to have a migrant background would be rated worse than the same dictation by a student without a migrant background. We assumed that there would be a statistically significant three-way interaction between migrant background × performance level × implicit attitude. In fact, a negative implicit attitude toward the performance of individuals with a migrant background was expected to lead to worse grades than those awarded to students without a migrant background, especially in the poor performance level condition, in which negative performance stereotypes are confirmed. Positive attitudes should lead to fewer differences between the student believed to have a migrant background and the student without a migrant background. For the average performance level, we expected fewer differences between the two students and the shape of implicit attitudes.

For the dependent variable *counted errors*, we expected only the performance level to have a main effect. Thus, we expected statistically significant more errors to be counted in the dictation with poor performance level than in the dictation with an average performance level.

As the number of counted errors should be a rule-based judgment which offers little scope for interpretation and, therefore, a judgment in which implicit attitudes hardly play a role, we did not establish parallel hypotheses for this dependent variable.

## Materials and Methods

Participation in all the following studies was voluntary, and informed consent was obtained from all participants via online consent forms that were embedded in all surveys. Every participant had to agree to the following statement: “I hereby confirm that I am of age, that I have read the consent form, and that I agree to take part in this study under the described conditions.” Participants were assured that all of their responses would remain confidential and they could stop filling in the questionnaire at any time. The studies were conducted in full accordance with the Ethical Guidelines of the German Association of Psychologists (DGPs) and the American Psychological Association (APA). At the time the data were acquired, it was not customary at most German universities to seek ethics approval for survey studies on such a subject. The study exclusively makes use of anonymous questionnaires. No identifying information was obtained from participants. We had no reason to assume that our survey would induce persisting negative states (e.g., clinical depression) in the participants.

### Development and Pre-testing of Material

#### Dictations to Operationalize the Performance Level

In order to develop material which allowed us to test our hypotheses, we decided to work with third graders’ dictations written in German. Such dictations allowed us to ask the participants to make both a less rule-based judgment, here by assigning a grade, as well as a rule-based judgment, such as counting the errors in the given dictation. Dictation is a common method for detecting spelling skills. The dictation task is used frequently in school practice (in Germany, but also, e.g., in the United States) to analyze students’ abilities to hear and record sounds in words (Adams and Bruck, 1995; Beck and Juel, 1995; Foorman et al., 1998). In school settings, it is especially used for beginning writers (Graham, 1982). In a dictation task, teachers read out a text and students have to write down what the teachers read. For our study, we needed dictations representing an objectively average and an objectively poor performance level. For this purpose, we chose two dictations with an average number of errors (5) and with many errors (30) respectively. The handwriting and content were held constant. These two dictations were pilot-tested with a sample of *N* = 45 pre-service teachers (74.5% female, *M* = 22.28 years old, *SD* = 2.84), who were shown the dictation in randomized order without any information about the student other than the fact that he or she was in third grade. They were asked to give a grade (based on the German grading system) between 0.75 (*very good*) and 6.00 (*very poor*) and to count the errors in the dictation. In the average performance condition, the participants counted *M* = 2.57 (*SD* = 1.42) errors, and in the poor performance condition they counted *M* = 26.28 (*SD* = 5.51) errors. In these two conditions, the number of errors counted differed statistically significantly [*t*(44) = 24.76, *p* < 0.001, *d* = -5.89]. The average performance was graded as *M* = 1.76, (*SD* = 0.50), whereas the bad performance was rated as *M* = 4.85 (*SD* = 1.16). The assigned grades were statistically significantly different from each other [*t*(44) = -16.32, *p* < 0.001, *d* = -3.46].

#### Names to Operationalize the Migrant Background

In order to test our procedure, which involved providing information about the migrant or non-migrant background of students, we pretested Turkish and German names. The sample size included *N* = 52 pre-service teachers (77.4% female, *M* = 22.04 years old, *SD* = 2.82). They were asked to rate different names with regard to their association with gender (1 = *female*, 2 = *male*, 3 = *no decision*) and with regard to their the association with a Turkish or German background (1 = *German*; 2 = *Turkish*; 3 = *no decision*). Furthermore, the pre-service teachers rated the names in terms of attractiveness (1 = *not attractive at all*; 7 = *very attractive*) and intelligence (1 = *not intelligent at all*; 7 = *very intelligent*).

The two names selected for our main study, Max and Murat, were each rated as names that are either highly associated with a German background (to operationalize the student without a migrant background: Max, German = 98.1%, no decision = 1.9%) or a Turkish background (to operationalize the student with a migrant background: Murat, German = 1.9%, Turkish = 98.1%). In terms of the variable gender, all participants rated the two names as being the names of a male student. These two names were associated with the lowest differences in assumed intelligence and attractiveness.^1^

#### Material for Measuring Implicit Attitudes

In order to assess participants’ implicit attitudes, we developed an Implicit Association Test (IAT) which measured the implicit performance-related associations concerning individuals with a migrant background. For the IAT, we used photos as target stimuli and performance-related adjectives as attribute stimuli. The selection of photos was based on a pretest with *N* = 111 participants (66.7% female, *M* = 27.94 years old, *SD* = 11.07). Each photo was a biometric passport photograph of a person. Participants were asked to rate 222 photos of male and female individuals with and without a migrant background in terms of the migrant background (1 = *German*; 2 = *Turkish*; 3 = *no decision*), attractiveness (1 = *not attractive at all*; 7 = *very attractive*), and intelligence (1 = *not intelligent at all*; 7 = *very intelligent*) of the individuals in the photos. Twenty-four photos of the most Turkish-looking and 24 of the most German-looking individuals (half female, half male) rated photos were selected (between 79.3% and 96.4% acceptance for the Germans and 80.5% and 91.9% for the Turkish-looking individuals) out of the 222 photos of male and female individuals of either a Turkish migrant background or German origin.

Additionally, 24 positive and 24 negative words related to performance were chosen. The words were presented to *N* = 52 pre-service teachers (72.2% female, *M* = 22.09 years old, *SD* = 2.82). They were asked to evaluate the word valence in relation to performance on a 7-point Likert-type scale, ranging from *connected to performance* to *not at all connected to performance*, as well as *related to very bad performance* to *related to very good performance*. We chose only words that were closely connected with performance (*M* from 5.75 to 6.81, *SD* from 0.44 to 0.88), and from these words, we chose the ones most associated with either a bad performance (24 words, *M* from 1.87 to 2.55, *SD* from 0.83 to 0.99) or good performance (24 words, *M* from 5.62 to 6.17, *SD* from 0.73 to 0.88).

### Participants

The participants in this study were 203 pre-service teachers (69.3% female) who were enrolled in a teacher training program at a university of education in Germany. They had a mean age of 23.39 (*SD* = 3.42) and a mean teaching experience of 2.12 months (*SD* = 12.21). All pre-service teachers were German and German native speakers. Within this sample, 86.8% of them had already successfully completed a school teaching internship as a mandatory part of their program.

The participants were recruited via notices posted on campus and through personal contacts. They received three Euros and chocolate for participating.

### Procedure and Design

The study had a 2×2 factorial between-subject experimental design. The participants received a dictation which was either average or poor (factor: performance level) and which was supposedly written by a student with or without a migrant background (factor: migrant background). The participants’ implicit associations were assessed as a continuous variable.

The participants were recruited through personal contacts at a university of education. After arriving in the lab, the participants were seated in front of a computer screen. They were informed that they were participating in a study to evaluate how important different teacher variables (e.g., experience or subject) are for performance assessment. At first, they had to fill in information about their demographics. Afterward, one dictation was shown and a brief introduction to the student who had allegedly written the dictation was given. The participants were asked to rate the performance of the shown dictation by giving it a grade and counting the number of errors (dependent variable). The participants could enter the errors and the grade in an open field. (“How many mistakes did the dictation have?” and “What grade would you award the student for this dictation?”). They were asked to apply the German grading system (range from 0.75 to 6.00 with 0.75 indicating the best performance and 6 the worst performance) when grading and to count the mistakes in terms of the errors they found. Please note that, given this coding of the grades, high values for the variable *grade* represented a low performance assessment.

After finishing the assessment, they were asked (as a manipulation check) what the name of the student was and how old the student was. In a last step, they completed the IAT. After completing the IAT, they were given feedback regarding how experienced teachers rated the performance of the dictation. Then they were given their reward and thanked. The participants could voluntarily sign up to receive an email informing them on the purpose of the study. Participants who signed up received an email after the study in which they were informed that the study examined the differences in the grading of a dictation depending on the assumed migrant background of the students, and they were informed about the IAT task.

Participants were randomly assigned to the experimental conditions; *N*: 50 participants had to rate a student with a supposedly migrant background and an average performance level. There were 51 participants in all other conditions.

### Instruments

#### Migrant Background

The participants were told that the dictation had been written by a male student with or without a migrant background (operationalized by the pretested names of the students: Max vs. Murat). To introduce the student to the participants in the condition *without a migrant background* (the verbalization for the condition *with a migrant background* can be found in brackets), they were given the following brief introduction:

“The following dictation was written by Max [Murat]. He is a third grade student and 8 years old.”

#### Performance Level

After reading the introduction, the participants were given the pre-tested dictation with either an average or poor performance [operationalized by a dictation, one with an average number of mistakes (5) versus one with many mistakes (30)].

#### Implicit Association Test

To measure the implicit attitudes, the IAT was used. As part of the IAT, participants had to complete 12 practice trials for the target concept (migrant background) and 12 practice trials for the attribute concept (connection to performance). Then they had to sort both together in 24 trials (training trials) to get to know the task. This was followed by 48 critical trials (measurement trials). Afterward, the categorization was changed. They had 12 practice trials for the new order of the categories on the screen and 24 practice trials for the new grouping. Then, as in the first step, 48 critical trials followed. Randomized photos and words were shown.

The reaction time was expected to be faster if highly associated categories (*stereotypic*; e.g., migrant background and dumb) shared a response key compared to when less associated categories (e.g., migrant background and intelligent) shared a key (Greenwald et al., 1998). Individuals who associate a migrant background with a lower performance ability should react more slowly in the *non-stereotypic* condition than in the *stereotypic* condition. It should be easier for them to sort the photos and words if the highly associated groups share a key.

The result is a measurement of the differential association between the target and attribute stimuli, in our case, the associations between performance and migrant or non-migrant background. The implicit associations were measured by a *d*-score, which was calculated on the basis of the improved scoring algorithm by Nosek et al. (2005).^2^ In our study, a *d*-score below zero indicated stereotypes in favor of the performance of individuals with a migrant background, which meant a higher association between individuals with a migrant background and high performance, and a *d*-score over zero indicated stereotypes in favor of performance of individuals without a migrant background and, therefore, a higher association between individuals without a migrant background and high performance.

### Statistical Procedure

In order to analyze the statistical effects of the migrant background, performance level, and implicit attitudes on the counted errors and the grade, we calculated linear regression analyses with three models for each dependent variable.^3^ The model M1 included all possible main effects (effects of performance level, migrant background, and implicit attitude). Model 2 (M2) included the two-way interaction effect between the performance level and migrant background, and between performance level and implicit attitude as well as the interaction term between migrant background and implicit attitude. The last model (M3) also included the three-way interaction between the three independent variables. We reported whether each model resulted in a statistically significant increment in explained variance. For each model, we analyzed whether the beta-coefficients for a main effect or an interaction effect were statistically significant. In the case of statistically significant interaction effects, we used simple slope analyses to investigate the nature of the interaction.

Given our hypotheses, we applied a 95% one-tailed confidence interval level.^4^

## Results

### Data Screening and Manipulation Check

Before going deeper into the analyses, we checked whether the manipulation of the *migrant background* worked. None of the participants associated a name with the wrong migrant background. In total, 88.8% could correctly assign the child’s name to the respective background (Turkish name vs. German name, depending on the condition). The remaining 11.2% of our test persons did not state anything.

For the 203 study participants, 195 IATs were evaluated. Of these, six individuals had to be excluded due to technical problems. Following the suggestions of Greenwald et al. (2003), two participants had to be excluded due to their response time or number of mistakes. The order of the blocks showed no differences concerning the *d*-score. Therefore, both conditions were evaluated jointly.

### Descriptives

See **Table 1**, **2** for the descriptives and correlations between the variables.

**Table 1:** Mean values and standard deviation (in brackets) of the grades and errors in the dictation, separated by the performance level and migrant background.

	Grade			Errors		
	*M* (*SD*)			*M (SD)*		
Performance level	No migrant background	Migrant background	*Cohen’s D*	No migrant background	Migrant background	*Cohen’s D*
Average	1.87 (0.46)	2.03 (0.60)	-0.30	4.76 (1.68)	5.08 (1.21)	-0.22
Poor	3.64 (1.20)	4.15 (1.10)	-0.44	29.40 (4.91)	29.68 (3.79)	-0.06
*Cohen’s D*	-1.95	-2.39		-6.71	-8.74	

*Mean and standard deviation (in brackets) of the d-score.*

*d-score = 0.30 (0.46).*

**Table 2:** Pearson correlation coefficients of all variables.

	Performance level	Migrant background	Implicit attitude	Grade	Errors
Performance level	1	–	–	–	–
Migrant background	0.005	1	–	–	
Implicit attitude	0.072	–0.048	1	–	–
Grade	0.731^∗∗^	0.121	–0.007	1	–
Errors	0.967^∗∗^	0.012	0.084	0.729^∗∗^	1

*^∗∗^p < 0.01; ^∗^p < 0.05.*

#### Implicit Association Test

On average, the implicit association (= *d*-score) was 0.30 (*SD* = 0.46), indicating that the overall implicit association of the participants was in favor of individuals without a migrant background. However, the standard deviation revealed substantial between-individual differences in implicit attitude.

#### Effect of Migrant Background, Performance Level, and Implicit Association on the Errors and the Grade

**Table 3** presents the unstandardized regression coefficients (*b*), the standard errors, and the standardized regression coefficients (β) for the two regressions predicting assigned grades and number of counted errors. We will first report the results for the dependent variable grade.

**Table 3:** Summary of regression analysis for variables predicting errors and grades.

		Grade			Errors		
Model	Variable	*b*	SE	β	*b*	SE	β
M1	PL	2.023**	0.126	0.755**	24.547**	0.478	0.965**
	MB	0.287*	0.126	0.107*	0.316	1.045	0.012
	IA	-0.164	0.137	-0.057	0.427	0.518	0.016
	*R*^2^	0.573			0.932		
M2	PL	1.647**	0.406	0.615**	24.343**	1.532	0.957**
	MB	-0.073	0.398	-0.027	0.578	1.516	0.023
	IA	1.107*	0.548	0.381*	-0.096	0.783	-0.004
	PL×MB	0.335	0.250	0.273	-0.054	0.959	-0.005
	PL×IA	-0.395	0.273	-0.221	1.353	0.834	0.071
	MB×IA	-0.464	0.273	-0.254*	-0.841	0.826	-0.044
	*R*^2^	0.583			0.932		
M3	PL	1.148*	0.474	0.428*	24.330**	1.550	0.956**
	MB	-0.536	0.457	-0.200	0.567	1.530	0.022
	IA	-1.274	1.304	-0.439	-0.106	0.798	-0.004
	PL×MB	0.660*	0.296	0.538*	-0.039	0.988	-0.003
	PL×IA	1.232	0.854	0.687	1.409	1.211	0.074
	MB×IA	1.158	0.852	0.634	-0.786	1.194	-0.041
	PL×MB×IA	-1.090*	0.543	-0.994*	-0.068	1.059	-0.006
	*R*^2^	0.590			0.932		

*The table displays the unstandardized regression coefficients (b), standard errors (SE), standardized regression coefficients (β), and the respective significance values (p). PL, performance level (0 = average, 1 = poor), MB, migrant background (0 = no migrant background, 1 = migrant background), IA, implicit attitude.*

*^∗∗^p < 0.01; ^∗^p < 0.05.*

#### Grades

The results for the main effects in Model 1 show that performance level, migrant background, and the implicit associations predicted 57.3% (adjusted *R*^2^) of the variance of the grade. The overall model was statistically significant [*F*(3,191) = 87.93, *p* < 0.001]. As can be seen from the regression coefficient in **Table 3**, grading of students’ dictation was significantly affected by the performance level (β *=* 0.76, *p* < 0.001). Better grades were assigned to dictations with an average performance level (*M* = 1.95, *SD* = 0.53) than the grades assigned to dictations with a poor performance level (*M* = 3.89, *SD* = 1.17). The difference added up to 2.023 grades. As can be seen from the regression coefficient, migrant background also affected the grades (β = 0.11, *p* < 0.05). The dictation was graded less favorably when a student was assumed to have a migrant background (*M* = 3.09, *SD* = 1.37) compared to a student without a migrant background (*M* = 2.76, *SD* = 1.27*).* The implicit attitudes had no statistically significant main effect on the grade (β = -0.16, *p* = 0.230*).*

Model 2 did not lead to a statistically significant increase in the explained variance of the grade [*F*(3,188) = 46.24, *p* = 0.061, *R*^2^ = 0.583, Δ*R*^2^ = 0.016]. However, none of the included two-way interaction terms were statistically significant.

Model 3 led to a statistically significant increase in the explained variance of the grade [*F*(1,187) = 40.85, *p* < 0.05, *R*^2^ = 0.590; Δ*R*^2^ = 0.009]. In this model, we also observed a significant two-way interaction between migrant background and performance level, which, however, was qualified by the predicted three-way interaction between migrant background, performance level, and implicit attitude, which was statistically significant (β = -0.99, *p* < 0.05).

##### Simple slope analysis

**FIGURE 1:**
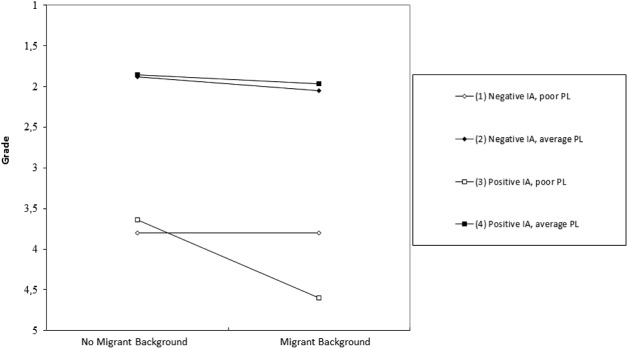
Grade as a function of migrant background, performance level (PL), and implicit attitude (IA). Coding: Positive IA = -1 SD, Negative IA = +1 SD.

In order to interpret the direction of the interaction effects, we used simple slope analyses (Aiken et al., 1991; Dawson and Richter, 2006). For the three-way interaction with implicit attitude as a continuous variable, the regression lines were plotted for one standard deviation above and below the mean of the *d*-score of the IAT (Aiken et al., 1991). The slope for the variable migrant background of a lower implicit association (higher association with individuals with a migrant background and good performance over individuals without a migrant background and high performance) and a bad performance was significantly more pronounced than the one for a high implicit association (higher association of individuals without a migrant background and high performance) and a bad performance (*t* = -2.65; *p* < 0.05). As **Figure 1** shows, the variation in migrant background resulted in more pronounced differences in the grade for dictations with a low performance level, as there were more positive associations with the performance level of individuals with a migrant background compared to when the implicit associations were more negative. However, the slope for more positive associations showed that, in these cases, migrant background was associated with poorer grades, which was not the case given more negative associations.

#### Number of Counted Errors

The results for the main effects in Model 1 show that performance level, migrant background, and implicit attitude predicted 93.2% (adjusted *R*^2^) of the variance of the errors. The overall model was statistically significant [*F*(3,189) = 884.76, *p* < 0.001]. As can be seen from the regression coefficient in **Table 3**, the performance level was statistically significantly affected by the number of errors counted in students’ dictations (β = 0.97, *p* < 0.001). Fewer errors were counted in dictations with an average performance level (*M* = 4.92; *SD* = 1.46) compared to the number of errors found in dictations with a poor performance level (*M* = 29.54, *SD* = 4.36). No statistically significant effect of the migrant background (*M_MB_* = 17.38, *SD* = 12.67; *M_nMB_* = 17.08, *SD* = 12.91; β = 0.01, *p* = 0.509) or of the implicit association (β = 0.02, *p* = 0.410) was found.

Model 2 led to no statistically significant increase in the explained variance of the counted errors [*F*(3,186) = 442.35, *p* = 0.43; *R*^2^ = 0.932, Δ*R*^2^ = 0.000]. None of the included two-way interaction terms were statistically significant (**Table 3**).

Model 3 also did not lead to a statistically significant increase in the explained variance of the counted errors [*F*(1,185) = 377.126, *p* = 0.949, *R*^2^ = 0.932, Δ*R*^2^ = 0.000]. The three-way interaction term was not statistically significant.^5^

## Discussion

This experimental study tested the influence of migrant background on assigned grades and counted the errors in a dictation. We assumed that students with a migrant background would be disadvantaged due to errors in the evaluation of students by teachers. This was assumed to be more likely when individuals make a less rule-based judgment, which demands them to make an extensive judgment, integrating different components into an overall judgment (here: grade). On the other hand, when making a rule-based judgment, it is less likely because this type of judgment requires one only to make a judgment on the basis of clearly defined rules without having to integrate different information. The latter leaves little space for integrating stereotypic information into the judgment. Therefore, this study investigated potential biases in the assignment of grades (as a less rule-based judgment) and the number of counted errors (as a rule-based judgment).

### Rule-Based Judgments

Our results support the hypothesis that the assignment of grades is more strongly influenced by information on the students’ migrant background than the number of counted errors. We found the predicted main effect of migrant background on the grade and a statistically significant three-way interaction between performance level, migrant background, and implicit attitude. On the other hand, we found a statistically significant main effect only for the number of counted errors on performance level – an effect which was also observed for the grades. We did not find a statistically significant main effect of the migrant background nor a statistically significant interaction effect with performance level or implicit association. This pattern of results indicates that biased performance assessment of students with a migrant background in comparison to students without a migrant background does not occur when the diagnostic process requires a rule-based judgment (e.g., counting errors), but rather when teachers have to make a less rule-based judgment (here: assigning grades).

### Effects of Performance Level and Migrant Background

As already mentioned, we proved a strong main effect of performance level on the grade. A poor performance in dictation was graded less favorably than an average performance. Overall, we can conclude that the performance level was properly assessed by the teachers. This leads to the conclusion that teachers can clearly differentiate between performance levels even when making a less rule-based rating. Furthermore, this finding shows that these ratings are valid (given that the effect of performance level is much stronger than the effect of migrant background).

However, when a student was assumed to have a migrant background, the dictation was graded less favorably compared to a student without a migrant background, namely by 0.3 grade steps. This main effect of migrant background indicates that the information about a migrant background makes a difference to the way the participant is assessed. It indicates that biased judgments are made when assessing students with a migrant background. Here, the teachers are biased when making a less rule-based judgment, whereas they are able to correctly count the errors when performing a rule-based rating without taking migrant background into account.

Despite the same number of counted errors, different grades were assigned depending on the background of the student. Therefore, the question arises as to why such differences might occur when teachers are clearly able to assess performance when applying a rule-based judgment but fail to transfer this when making the same, less rule-based judgment. One could assume that the teachers apply stricter standards to their students with a migrant background. Previous findings with respect to teacher expectations regarding the future performance of students suggests that judgments are not negatively biased, as anticipated, but are more accurate in comparison to judgments about students without a migrant background (Tobisch and Dresel, 2017). This indicates that teachers’ judgments are possibly positively biased for students without a migrant background and that they therefore tend to judge them mildly. Thus, the disadvantage of students with a migrant background would primarily be a milder evaluation of students without a migrant background and not a negative bias against students with a migrant background. It would be interesting for further research to examine whether differences in grading result from a positive or negative bias, or a combination of both.

### Effects of Implicit Attitudes

Two additional factors, which together could have an influence on judgments, may contribute to the different assessment of performance during grading: performance level and implicit attitudes. The difference in grades was more pronounced at a poor performance level than at an average performance level, but only when the implicit attitude of the individual making the assessment was taken into account. This shows the importance of implicit attitude for this effect, and points toward a use of stereotypes in the grading process.

Thus, migrant background has a stronger influence on grades at a poor performance level when implicit attitudes are taken into account. This might be because teachers make stereotype-based judgments. However, the results did not support the hypothesis that differences would be found especially in the dictation with a poor performance level *and* when the participant had a negative implicit attitude. This was expected because the bias might be more pronounced if the performance of a student matches the teacher’s attitude. Our findings related to the implicit attitudes suggest that grading of the poor performance level was contrary to the attitude of the participants. If we examine the alignment of these effects, the difference depending on performance level and migrant background was more pronounced when the participants associated high performance more strongly with individuals with a migrant background than with individuals without a migrant background. A positive attitude toward individuals with a migrant background led to *worse* grading than a negative attitude toward individuals without a migrant background. This interaction effect was surprising because we expected it to be in the opposite direction.

It remains an open question as to how to interpret this divergence between the hypotheses and findings. One reason could be that the teachers with a positive implicit attitude toward students with a migrant background who had to assess a student with a migrant background who performed poorly had high expectations because of their positive attitude toward students with a migrant background, and these expectations were disappointed by the dictation. Thus, they may have graded the dictation more severely because it did not fulfill their expectation. However, before putting forward alternative explanations, the unexpected direction of the interaction between performance level, migrant background, and implicit attitude should be replicated in future studies.

### Limitations

Some limitations of our research should be kept in mind. The teachers had to judge students based on limited information and without knowing about the students’ prior development at school. They were not provided with any additional information about the students. The external validity of the results might therefore be limited. However, an experimental approach, such as in the present study, has a clear benefit. Different grades can, of course, be rooted in actual performance differences. The experimental approach allowed us to control for such differences and helped us to clearly identify the sole effect of migrant background on the different types of performance rating. This is difficult to realize in field studies. Therefore, the experimental approach was more suitable for our purposes, even if there was some loss of external validity.

We operationalized migrant background in this study by using student names. The risk of doing this is that names might not only transfer information about migrant background, but also several other associations or assumptions. One of them might be socioeconomic status. As past research suggests, teachers rated students differently according to their names (Harari and McDavid, 1973; Dusek and Joseph, 1983; Oudman et al., 2018) and different names are connected to a varying degree with student characteristics, among other things, achievement and socioeconomic status.

Therefore, our results could be due to the impression of a migrant background, on the one hand, or due to associations with other student characteristics conveyed by the name, on the other hand, or at least to a combination of several characteristics. For this reason, we intentionally selected two names that strongly transport the respective backgrounds, and furthermore are comparable in terms of other variables (intelligence, attractiveness, gender). Our pretest revealed that none of the Turkish names tested were rated on an entirely comparable level to German names in terms of intelligence. All Turkish names were associated with relatively lower intelligence. This problem is reported in other studies, also with regard to socioeconomic status (Tobisch and Dresel, 2017). Apparently, there is a strong link between Turkish names and low socioeconomic status. This is not surprising because one of the stereotypes associated with Turkish individuals appears to be that they have lower abilities in terms of performance (Kahraman and Knoblich, 2000).

Nevertheless, we have to stress the fact that our findings might result from a combination of two effects: migrant background and lower socioeconomic status. Previous experimental and field studies have shown that the effects of migrant background are smaller when taking socioeconomic status into account, but that they do not disappear (Bonefeld et al., 2017; Tobisch and Dresel, 2017). Therefore, we conclude that our effects might be smaller, but would still exist if socioeconomic background were held constant. Confounding effects should be kept in mind in any case.

In our study, we only provided information about male students. They are more often judged less favorably than female students in terms of their work habits and rated as less competent than girls (Smith, 1988; Parks and Kennedy, 2007). Hence, stereotypical expectations concerning male students with a migrant background could be more pronounced than for female students. It is thus necessary to test whether the findings can be replicated for both female and male students. Therefore, future research should address the potential combined effects of migrant background and students’ gender on performance assessment.

With respect to implicit attitudes, we assumed that the IAT would be effective in measuring attitudes. On the one hand, previous studies have shown that attitudes can predict judgments and influence automatic and controlled behavior (van den Bergh et al., 2010). On the other hand, implicit attitudes have methodological advantages because measurement errors due to the interpretation of questions and response formats can be avoided. Additionally, an important point is that social desirability might play a role, especially when reporting attitudes toward individuals with a migrant background. Therefore, we collected only data on the implicit associations of the subjects. However, it might be of interest to capture also explicit attitudes in future research in order to draw comparisons between both measures.

### Implications

Our results have implications for teacher education. It should be kept in mind that, other than the variation of performance level, the dictation differed only with regard to the first name of the student. The information provided about the performance in the dictation was exactly the same for the different groups. Nevertheless, the dictation was graded differently depending on the background of the student. We can therefore infer that the biased judgments resulted from the variation in names. This highlights the importance of focusing on elucidation and teaching reasons for judgmental biases as well as strategies for avoiding biases in teacher education. Our results suggest that the difference in the assessment of students with and without a migrant background can be found in less rule-based judgments, whereas rule-based judgments are rather accurate, or at least do not differ depending on the background of the student. Based on this, we can clearly recommend incorporating fixed grading rules into teacher education. Pre-service and in-service teachers should be encouraged to prepare grading schemes on the basis of rule-based judgments (which they can clearly deduce) and to award grades according to these fixed standards. For example, when grading a dictation, they could make grading dependent on the number of errors found. Broadly speaking, (pre-service) teachers should define which rule-based judgments compose their less rule-based judgment in terms of overall judgment. Then they should decide on which of these rule-based judgments their judgment (e.g., grade) is based. By means of this decision, an integrative rule for awarding certain grades could be made. In this way, biases could be reduced.

From a practical perspective, judgment errors are of great importance when one considers that school grades in elementary school influence future school careers and also have a great influence on later educational access, such as admission to different courses or different careers (Glock and Krolak-Schwerdt, 2013). Thus, it is necessary that teacher judgments are as accurate as possible to ensure the best possibilities for all students.

Apart from this, it is important to say that the processes that lead to judgmental biases, such as using stereotypes as information when assessing performance, are often unconscious and not intentional. The use of stereotypical attitudes can save cognitive capacity (Macrae and Bodenhausen, 2000) and is therefore efficient. Nevertheless, precisely because these processes are often unconscious and unintentional, it is important to determine the mechanisms behind these judgment processes which can have a considerable influence on students.

## Author Contributions

All listed authors contributed meaningfully to the paper. MB developed the study concept. All authors contributed to the study design and analyzed and interpreted the data. MB prepared the draft manuscript, and OD provided critical revisions. All authors have approved the final version to be published and agree to be accountable for all aspects of the work and ensure that questions related to the accuracy or integrity of any part of the work are appropriately investigated and resolved.

## Conflict of Interest Statement

The authors declare that the research was conducted in the absence of any commercial or financial relationships that could be construed as a potential conflict of interest.

## Footnotes

1 All Turkish names were associated with lower ratings of intelligence compared to German names. This might reflect the stereotype stated above that Turkish individuals are associated with fewer performance-related variables. We tried to address this fact by using two names with a smaller difference with regard to this variable, but which still offer clear categorization of migrant or non-migrant background and gender.

2 Half of the participants had a version of the IAT in which the stereotypic pairing took place first and the non-stereotypic afterward, and the other half vice versa. This was the case when facing influences of the order. The order had no effect on the *d*-score and no effect on the regression models.

3 We decided to use regression analyses instead of an ANOVA to analyze the data because one of the variables was non-categorial (implicit association).

4 All reported effects remain statistically significant if two-tailed testing is used.

5 All the reported effects remain stable if several experience factors are controlled for, such as professional experience in teaching, experience in the assessment of dictations, number of terms, type of school they will teach at in the future, or if they already had their orientation course in school.
